# Safe laparoscopic resection of a giant schwannoma in the retroperitoneum using a fluorescent ureteral stent: a case report

**DOI:** 10.1093/jscr/rjab350

**Published:** 2021-08-31

**Authors:** Masato Nishimuta, Yorihisa Sumida, Masato Araki, Kouki Wakata, Kiyoaki Hamada, Tota Kugiyama, Ayako Shibuya, Shintaro Hashimoto, Keisuke Ozeki, Shigeyuki Morino, Soichiro Kiya, Masayuki Baba, Akihiro Nakamura

**Affiliations:** Department of Surgery, Sasebo City General Hospital, 9-3 Hirase, Sasebo, Nagasaki 857-8511, Japan; Department of Surgery, Sasebo City General Hospital, 9-3 Hirase, Sasebo, Nagasaki 857-8511, Japan; Department of Surgery, Sasebo City General Hospital, 9-3 Hirase, Sasebo, Nagasaki 857-8511, Japan; Department of Surgery, Sasebo City General Hospital, 9-3 Hirase, Sasebo, Nagasaki 857-8511, Japan; Department of Surgery, Sasebo City General Hospital, 9-3 Hirase, Sasebo, Nagasaki 857-8511, Japan; Department of Surgery, Sasebo City General Hospital, 9-3 Hirase, Sasebo, Nagasaki 857-8511, Japan; Department of Surgery, Sasebo City General Hospital, 9-3 Hirase, Sasebo, Nagasaki 857-8511, Japan; Department of Surgery, Sasebo City General Hospital, 9-3 Hirase, Sasebo, Nagasaki 857-8511, Japan; Department of Surgery, Sasebo City General Hospital, 9-3 Hirase, Sasebo, Nagasaki 857-8511, Japan; Department of Surgery, Sasebo City General Hospital, 9-3 Hirase, Sasebo, Nagasaki 857-8511, Japan; Department of Surgery, Sasebo City General Hospital, 9-3 Hirase, Sasebo, Nagasaki 857-8511, Japan; Department of Surgery, Sasebo City General Hospital, 9-3 Hirase, Sasebo, Nagasaki 857-8511, Japan; Department of Surgery, Sasebo City General Hospital, 9-3 Hirase, Sasebo, Nagasaki 857-8511, Japan

## Abstract

Schwannomas that occur in the retroperitoneal cavity are rare. We herein report a patient who underwent safe laparoscopic resection by using a preoperative 3D computed tomography (CT) image and a fluorescent ureteral stent during the surgery.

A 47-year-old man presented with left lower abdominal pain. CT showed a 10-cm continuous retroperitoneal tumor originating at the third lumbar nerve in the lower left abdomen. Schwannoma was suspected. We underwent laparoscopic resection of the tumor guided by 3D images obtained preoperatively. A fluorescent ureteral stent was implanted during the surgery to improve visibility and protect the left ureter. The resection was completed without injury of other organs and vessels. The patient was discharged on postoperative Day 5.

By performing a preoperative simulation using 3D CT images, we could anticipate the anatomical findings and easily identify them intraoperatively. In addition, the fluorescent ureteral stent provided visual support, thereby contributing to safe surgery.

## INTRODUCTION

Schwannomas originate at peripheral nerve sheaths [1]. Those that occur in the retroperitoneal cavity are rare, accounting for only 0.75–2.6% of all retroperitoneal tumors [2, 3]. Schwannomas are mostly benign and are associated with no characteristic clinical findings. They are often diagnosed only because they are large enough to cause abdominal symptoms. Because some schwannomas have a malignant course [1]; however, resection is deemed necessary. In such cases, it must be recognized that there are multiple important vessels and organs in the retroperitoneum, so great care must be taken during the resection. We report a patient who underwent safe laparoscopic resection after we constructed a preoperative 3D computed tomography (CT) image for intraoperative guidance as well as implanting a fluorescent ureteral stent during the surgery to improve visibility.

## CASE REPORT

**Figure 1:**
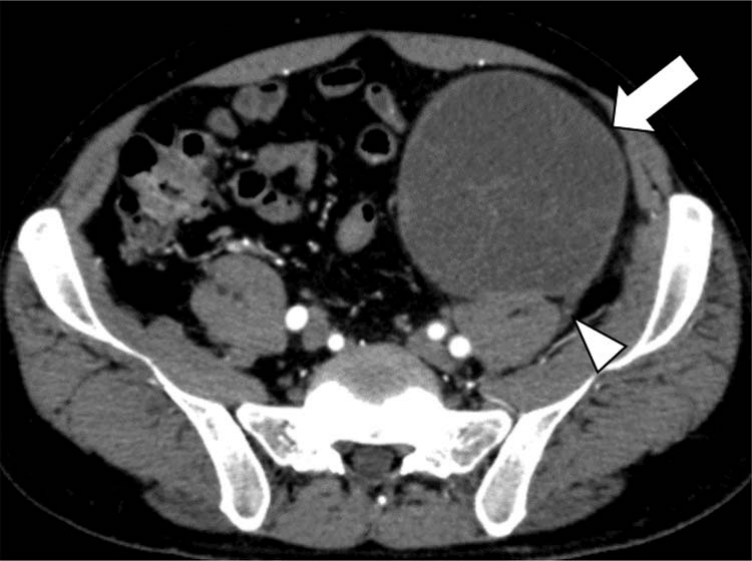
Contrast-enhanced CT confirmed a 10-cm hypodense solid mass (arrow) in the retroperitoneum of the lower left abdomen. It showed that the tumor was contiguous with the third lumbar nerve (arrowhead), suggesting that it was a schwannoma.

A 47-year-old man was admitted to his local hospital complaining of left abdominal pain. CT showed a 10-cm tumor in the left abdomen, and he was referred to our hospital. Contrast-enhanced CT confirmed a 10-cm hypodense solid mass in the retroperitoneum of the lower left abdomen. It showed that the tumor was contiguous with the third lumbar nerve, suggesting that it was a schwannoma (Fig. 1). Because the tumor was huge and its presence seemed to be associated with abdominal symptoms, we planned to resect it via less-invasive laparoscopic surgery.

**Figure 2:**
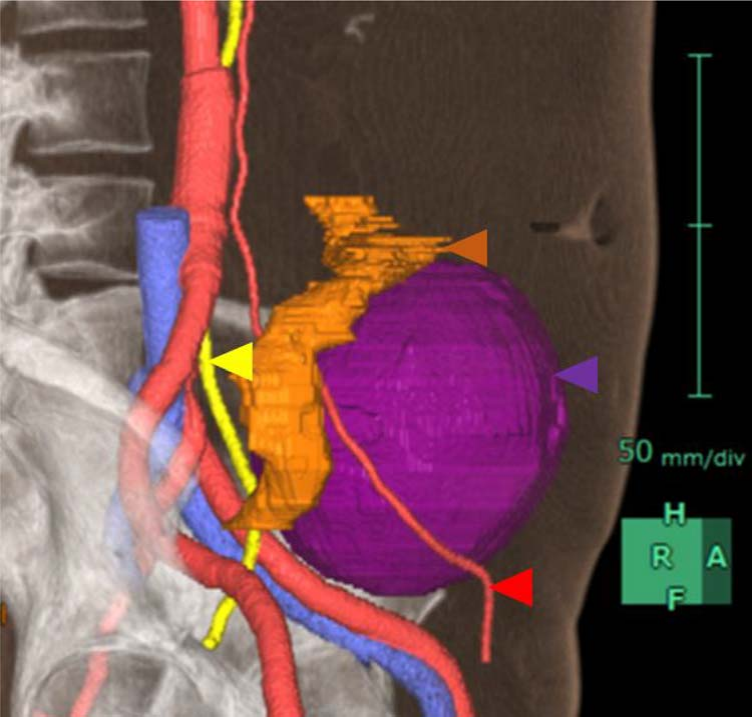
3D image constructed based on CT scans. Purple area is the tumor, yellow area is the left ureter, red areas are the left testicular arteries and orange area is the sigmoid colon.

As the tumor was close to the left ureter and testicular arteries and veins, there was concern about their injury. Hence, we constructed a 3D CT image preoperatively to understand its anatomical positioning and surroundings (Fig. 2). In addition, to avoid left ureter injury, we placed a fluorescent stent in the left ureter preoperatively.

**Figure 3:**
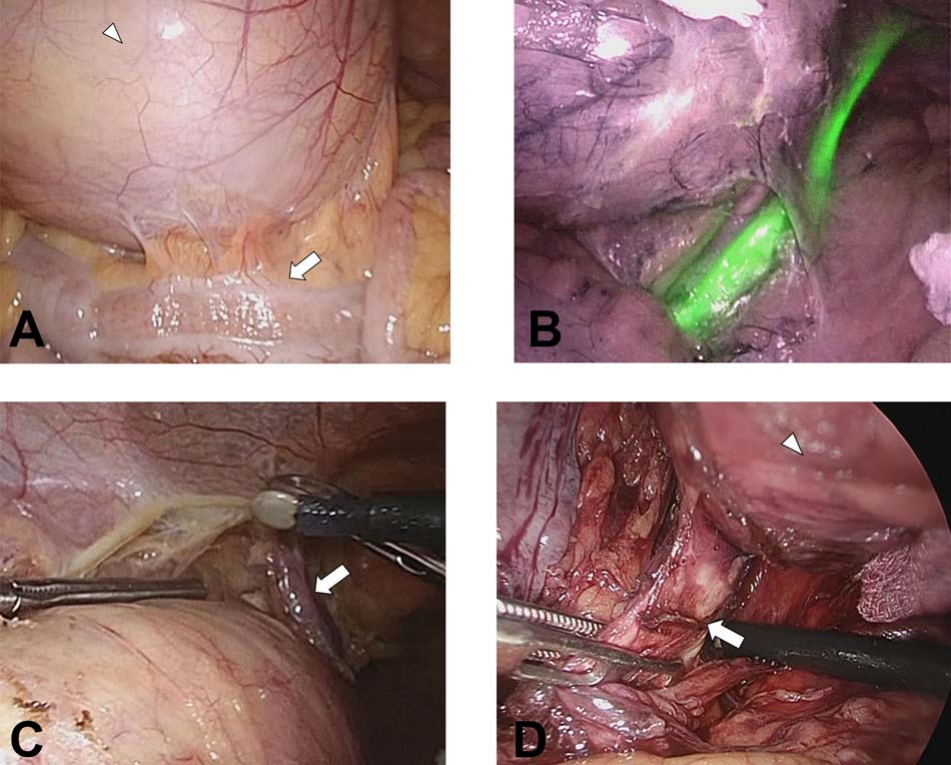
(**A**) The tumor (white arrowhead) was on the dorsal side of the sigmoid colon (white arrow) in the lower left abdomen. (**B**) Left ureter is clearly recognized using an infrared imaging videoscope. (**C**) The left testicular arteries and veins (white arrow) could be separated without injury. (**D**) The third lumbar nerve (white arrow) continuous with the tumor (white arrowhead).

**Figure 4:**
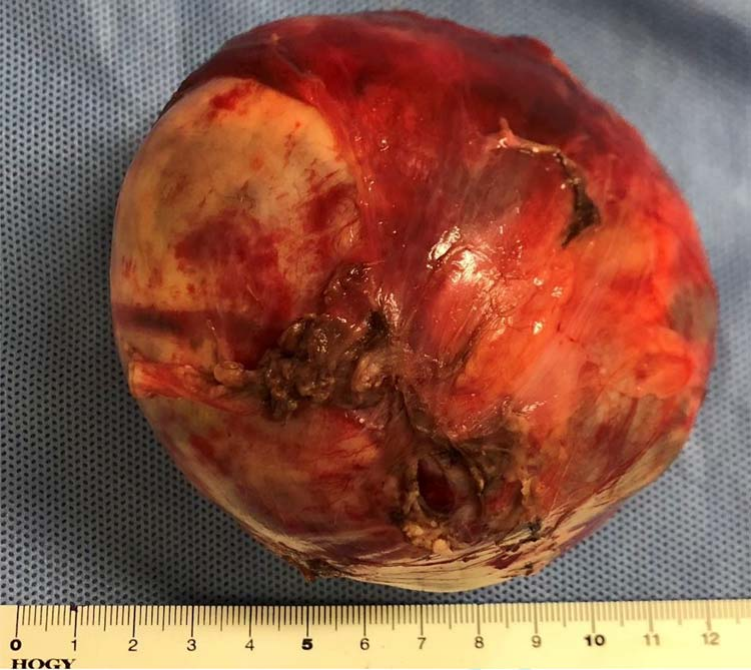
Macroscopic findings of the tumor.

**Figure 5:**
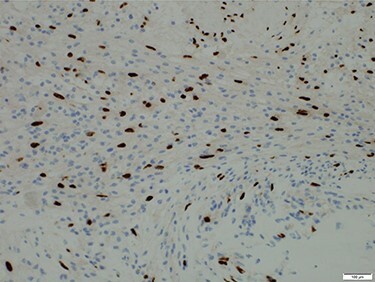
Microscopic findings of the tumor. Immunostaining for S-100 protein was positive.

We then undertook five-port laparoscopic surgery using an infrared imaging videoscope. Two 10-mm ports were placed on the cranial side of the umbilicus and in the lower right abdomen, respectively, and three 5-mm ports were placed, respectively, on the left and right upper abdomen and the cranial side of the pubis. The tumor was on the dorsal side of the sigmoid colon in the lower left abdomen (Fig. 3a). We could then perform the dissection along the tumor, clearly visualizing the left ureter (Fig. 3b) and the left testicular arteries and veins (Fig. 3c). Finally, the third lumbar nerve continuous with the tumor was dissected near the tumor (Fig. 3d), and the tumor was removed from an umbilicus wound that extended 7 cm. Total operative time was 248 min, with an estimated blood loss of 29 ml. The surgical specimen was soft and spherical, with a capsule of 10 cm maximum diameter. The specimen contained a septum that was filled with a dark-red serous fluid (Fig. 4).

Postoperatively, the patient experienced femoral nerve palsy, but it diminished over time because of rehabilitation. He was discharged from the hospital on postoperative Day 6.

Histologically, the tumor showed irregular formation with spindle cells and foci of foam cells. Immunostaining for S-100 protein was positive (Fig. 5). Based on these findings, schwannoma was diagnosed.

Currently an outpatient, he is free from abdominal symptoms. We plan to follow him with CT imaging to check for signs of recurrence.

## DISCUSSION

Schwannomas occur only rarely in the retroperitoneal cavity and are predominantly benign. Previous reports indicate that schwannomas appear at incidences of 44.8% in the head and neck, 19.1% in the upper limbs, 13.5% in the lower limbs [1] and 0.75–2.6% in the retroperitoneum [2, 3]. Retroperitoneal schwannomas are often huge because they produce no characteristic symptoms and become apparent only when they become large enough to produce compression symptoms such as abdominal pain and abdominal discomfort [4, 5]. These very large schwannomas may undergo cystic degeneration because of hemorrhage/necrosis indicative of the longstanding nature of the lesion. These tumors are called ‘ancient’ schwannomas [6].

Most schwannomas are benign and have a good prognosis. Others, however, have a malignant course that requires patient monitoring after the lesion’s resection because of high relapse rates [1]. Resection is thus often selected for large schwannomas to alleviate the patient’s compression-induced symptoms and to confirm the diagnosis. Because the retroperitoneum contains major vessels as well as the duodenum, kidneys and ureters; therefore, great care must be taken during retroperitoneal tumor resection. In the patient reported herein, the left ureter and left testicular arteriovenous vessels were close to the tumor. We overcame this difficulty, however, by constructing preoperative 3D CT images that helped us understand and navigate the patient’s specific anatomy. The 3D images also helped us determine proper port placement.

In recent years, there have been some reports on the usefulness of laparoscopic surgery for retroperitoneal tumors [7–9]. In addition, a fluorescent ureteral stent has been developed that improves visibility, thereby preventing ureteral injury [10]. By placing a fluorescent ureteral stent preoperatively and performing laparoscopic surgery with an infrared imaging videoscope, we could preserve the ureter more reliably. After searching a PubMed database on the subject, we believe that this report of a case of laparoscopic retroperitoneal tumor resection with a fluorescent ureteral stent is unique. We are currently considering the possibility of active use of fluorescent ureteral stents during laparoscopic surgery in patients with advanced colorectal cancer.

In conclusion, we suggest the usefulness of preoperative 3D imaging to obtain 3D guidance images for upcoming laparoscopic surgery to resect huge retroperitoneal schwannomas. We also suggest that the use of intraoperative fluorescent ureteral stents, which improve visibility, could allow safer laparoscopic surgery.

## Data Availability

The data used in the current study are available from the corresponding author on request.

## References

[1] Cury J, Coelho RF, Srougi M. Retroperitoneal schwannoma: case series and literature review. Clinics (Sao Paulo) 2007;62:359–62. 17589680 10.1590/s1807-59322007000300024

[2] Wong CS, Chu TYC, Tam KF. Retroperitoneal schwannoma: a common tumour in an uncommon site. Hong Kong Med J 2010;16:66–8. 20124578

[3] Okuyama T, Tagaya N, Saito K, Takahashi S, Shibusawa H, Oya M. Laparoscopic resection of a retroperitoneal pelvic schwannoma. J Surg Case Rep 2014;2014:rjt122. 24876325 10.1093/jscr/rjt122 PMC3895046

[4] Daneshmand S, Youssefzadeh D, Chamie K, et al. Benign retroperitoneal schwannoma: a case series and review of the literature. Urology 2003;62:993–7. 14665342 10.1016/s0090-4295(03)00792-1

[5] Goh BKP, Tan YM, Chung YF, Chow PKH, Ooi LLP, Wong WK. Retroperitoneal schwannoma. Am J Surg 2006;192:14–8. 16769268 10.1016/j.amjsurg.2005.12.010

[6] Theodosopoulos T, Stafyla VK, Tsiantoula P, et al. Special problems encountering surgical management of large retroperitoneal schwannomas. World J Surg Oncol 2008;6:107. 18834531 10.1186/1477-7819-6-107 PMC2567322

[7] Zhang L, Gao M, Zhang T, et al. Surgical management of retroperitoneal schwannoma complicated with severe hydronephrosis: a case report. Medicine (Baltimore) 2018;97:e12528. 30278542 10.1097/MD.0000000000012528 PMC6181516

[8] Ribeiro MA Jr, Elias YG, SdeS A, et al. Laparoscopic resection of primary retroperitoneal schwannoma: a case report. World J Clin Cases 2020;8:4114–21. 33024769 10.12998/wjcc.v8.i18.4114 PMC7520759

[9] Hajimohammadi A, Kermansaravi M. Laparoscopic approach for the diagnosis and treatment of retroperitoneal schwannoma. J Res Med Sci 2020;25:100. 33273945 10.4103/jrms.JRMS_510_20 PMC7698375

[10] White LA, Joseph JP, Yang DY, et al. Intraureteral indocyanine green augments ureteral identification and avoidance during complex robotic-assisted colorectal surgery. Colorectal Dis 2021;23:718–23. 33064915 10.1111/codi.15407

